# Protection Against CNS-Targeted Rabies Virus Infection is Dependent upon Type-1 Immune Mechanisms Induced by Live-Attenuated Rabies Vaccines

**DOI:** 10.3390/tropicalmed2030022

**Published:** 2017-07-04

**Authors:** Aurore Lebrun, Samantha Garcia, Jianwei Li, Rhonda B. Kean, D. Craig Hooper

**Affiliations:** 1 Department of Cancer Biology, Thomas Jefferson University, Philadelphia, PA 19017, USA; aurore.lebrun@jefferson.edu (A.L.); samantha.garcia@jefferson.edu (S.G.); jianwei.li@jefferson.edu (J.L.); rhonda.kean@jefferson.edu (R.B.K.); 2 Department of Neurological Surgery, Thomas Jefferson University, Philadelphia, PA 19017, USA

**Keywords:** rabies, vaccine, type-1 immunity, type-2 immunity

## Abstract

Rabies remains a major public health issue worldwide, especially in developing countries where access to medical care can represent a real challenge. While there is still no cure for rabies, it is a vaccine-preventable disease with pre- and post-exposure prophylaxis regimens approved by the World Health Organization (WHO). However, many rabies-exposed individuals have limited access to vaccines and virus-neutralizing antibodies approved for post-exposure prophylaxis. Unfortunately, any delay in the administration of these reagents can have lethal consequences. This highlights the need to develop cost-effective immunological reagents with a greater window of efficacy. Live-attenuated vaccine strains of rabies virus presents a potential treatment in filling this gap. We show here that immunization with live-attenuated vaccines provide long-lasting rabies immunity, superior to the protection induced by inactivated vaccines. In the absence of an immunostimulatory adjuvant, vaccination with multiple doses of inactivated rabies virus induces a type-2 immune response. This type of immunity is highly effective at inducing neutralizing antibody but has limited efficacy in clearing the virus from central nervous system (CNS) tissues. In contrast, a single infection with live-attenuated rabies vaccine safely drives a type-1 immune response, associated with both the production of a neutralizing antibody and the clearance of wild-type rabies virus from CNS tissues. These results indicate that live-attenuated rabies strains have the potential to be more effective in post-exposure prophylaxis than conventional inactivated vaccines.

## 1. Introduction

Rabies is a central nervous system (CNS) disease, nearly always fatal for humans and most mammals, caused by host infection with the rabies virus (RABV). RABV is a single-stranded, negative sense, neurotropic RNA virus that belongs to the *Lyssavirus* genus of the *Rhabdoviridae* family [1]. Despite advances in the control of animal reservoirs and in human prophylaxis, rabies still accounts for over 60,000 human deaths worldwide, with most cases recorded in Asia and Africa [2]. Although incurable once clinical symptoms appear [3], rabies is largely preventable through mass vaccination of dogs in rabies-enzootic regions, which aims to eliminate the virus at its source [4], or via the use of anti-rabies biologics in humans after exposure [5]. Since Pasteur’s development of the first rabies vaccine in 1885, rabies prevention has evolved in two directions: pre- and post-exposure (PEP) prophylaxis. Pre-exposure prophylaxis, which involves a series of three or more intramuscular (i.m.) injections of inactivated rabies vaccine at 0, 7, and 28 days [6,7], is given only to at-risk populations such as veterinarians, laboratory workers, and travelers to rabies endemic regions [8]. PEP, which consists of proper wound cleaning, immunization with an inactivated rabies vaccine, and injection of rabies immunoglobulins at the site of infection [8], is effective at preventing the development of the disease when administered to the patient within a short window after exposure to a suspected rabid animal [9].

Unfortunately, a large number of rabies-exposed patients fail to receive adequate PEP, primarily in resource-poor countries, largely due to the high cost or unavailability of rabies vaccine or rabies immunoglobulins [10]. The difficulties of rabies management in animal reservoirs and the inability to follow World Health Organization recommendations for PEP best practice in under-developed countries drives the need to improve the availability of safe, cost-effective rabies reagents [11]. Foremost among these advancements are both the use of human monoclonal rabies virus neutralizing antibodies in replacement of serum-based antibodies and new vaccine approaches that provide the critical, safe, and rapid induction of long-lasting immunity. The use of adjuvants in combination with inactivated RABV vaccines or infection with live-attenuated RABV vaccine strains are two strategies that may more rapidly induce rabies immunity, although it is now known that the nature of the immune response is also an important consideration. We previously reported that the outcome of RABV infection of neural tissues is dependent upon two key processes: (1) the early control of virus replication by IFN-γ-promoted innate immune mechanisms [12,13]; and (2) virus clearance from CNS tissue by the infiltration of immune effectors and the local production of virus-neutralizing antibodies (VNA) [14]. A rabies-specific immune response biased toward type-1 immunity (Th1 CD4 T cell response) is critical for both of these processes [15].

The present study is aimed at evaluating the efficacy in mice of live-attenuated RABV vaccine strains in triggering long-lasting immunity and protection against challenge with lethal wild-type RABV via routes distal from and proximal to the CNS. Several vaccine and wild-type RABV strains were used in various mice strains to take into account genetic variability in immunity. The immune response to live-attenuated RABV was compared to inactivated RABV, which more closely resembles current approved vaccines for humans [5]. We found that live-attenuated RABV strains consistently outperform inactivated vaccine strains, including a current commercial vaccine IMOVAX^®^, in the induction of protection against challenge with a lethal RABV. We show that this is largely due to differences in the class of immune response elicited: type-1 by live-attenuated RABV, and type-2 by inactivated RABV.

## 2. Materials and Methods

### 2.1. Mice and Study Approval

C57BL/6 and Swiss Webster mice (6 to 8 weeks of age) were purchased from Jackson Laboratory (Bar Harbor, ME, USA) and Taconic Biosciences (Germantown, NY, USA), respectively. All animals were monitored for survival and blood samples were collected at various times post-infection for serum antibody titers. All procedures were conducted in accordance with Public Health Service Policy on Humane Care and Use of Laboratory Animals under protocols approved by the Institutional Animal Care and Use Committee of Thomas Jefferson University (Animal Welfare Assurance Number A3085-01).

### 2.2. Virus and Vaccine Strains

SPBN Double GAS (GG) and SPBN Triple GAS (GGG), recombinant RABV vaccines containing two or three copies of a mutated glycoprotein, respectively, were developed as previously described [16,17]. Both the dog rabies virus 4 (DRV4) and the silver-haired bat rabies virus 17 (SHBRV-17) are highly pathogenic RABV strains of dog and bat origins. These lethal viruses were isolated from the brains of human victims and propagated in suckling mice brain [18]. The challenge virus standard F3 (CVS-F3) is an antibody escape-attenuated mutant, of dog origin, that differs from its parental strain by a single point mutation in its glycoprotein in position 333 [19]. IMOVAX^®^ is a licensed human diploid cell vaccine (Sanofi Pasteur, Swiftwater, PA, USA) prepared from the Pitman-Moore strain of RABV grown on MRC-5 human diploid cell cultures, in use in the USA since 1980 [20,21].

### 2.3. Virus Inactivation

IMOVAX^®^ is a commercially available vaccine inactivated with beta-propiolactone (BPL), an alkylating agent reacting with nucleic acids and proteins, widely used for the inactivation and increased safety of biological reagents such as viruses [22]. In some experiments, IMOVAX^®^ was reconstituted according to manufacturer’s directions and stored at either 25 °C or 40 °C for three weeks prior to use. The live-attenuated vaccine strains used in this study were inactivated either by exposing the virus to UV radiation for 45 minutes or by treatment with BPL (1:100 dilution) overnight at 4 °C.

### 2.4. Immunization and Challenge of Mice

A variety of immunization strategies were used and are detailed in each figure legend. Briefly, mice were immunized with 1 × 10^5^–1 × 10^8^ focus-forming units (f.f.u.) of live-attenuated or inactivated (f.f.u. determined before inactivation) RABV via intramuscular (gastrocnemius, i.m.g., 50 µL), intranasal (i.n., 10 µL) or intracranial (i.c., 5 µL) routes. Mice immunized with IMOVAX^®^ received 0.125–0.25 International Units (IU) of rabies antigen. Mice were challenged at 21–63 days post-immunization with 1 ×1 0^3^–1 × 10^5^ f.f.u. lethal RABV via one of the same routes as immunization.

### 2.5. Neutralizing Antibody (VNA)Titer and Serum Antibody Isotyping

Virus neutralizing antibody (VNA) titer was evaluated by the rapid fluorescent focus inhibition test as previously described [23]. The antibody isotypes were determined by ELISA using UV-inactivated Evelyn-Rokitnicki-Abelseth (ERA) virus as coating antigen and mouse-specific total IgG, IgG1, and IgG2a secondary antibodies, as described previously [24].

### 2.6. StatisticalAnalysis

Statistical significance of survival rates or IgG levels were compared with a one-way ANOVA followed by the Bonferroni multiple comparison test. Graphs were created and statistical analysis was performed using GraphPad Prism 5.0 software (La Jolla, CA, USA). Statistically significant differences between groups are denoted as follows: * *p* ≤ 0.01, ** *p* ≤ 0.005, and *** *p* ≤ 0.001.

## 3. Results

### 3.1. Live-Attenuated RABV are Protective and Stable at Room Temperature

**Figure 1:**
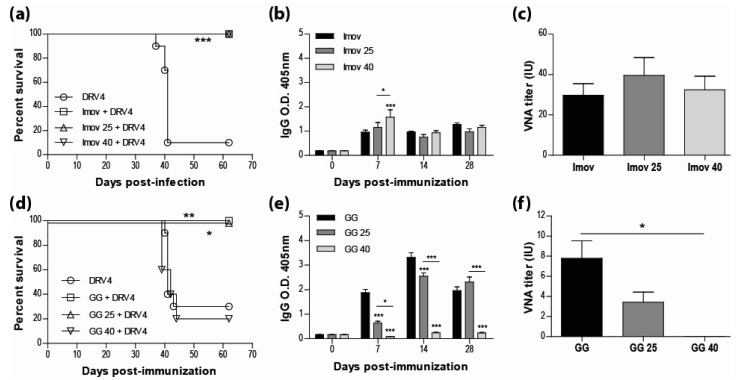
Live-attenuated RABV vaccines, stored at room temperature, retain sufficient infectivity to induce fully protective immunity. Swiss Webster mice were immunized with 0.25 IU of IMOVAX^®^ (**a**) or 1 × 10^5^ f.f.u. of GG (**d**) i.m.g. stored at various temperatures then challenged 30 days later with 1 × 10^5^ f.f.u. of DRV4 i.m.g. and monitored for survival. Data are expressed as percent survival (*n* = 5–10 per group). (**b**,**e**) Virus-specific Ab response for total IgG was determined by ELISA at 7, 14 and 28 d.p.i. for all groups (dilution 1:40). (**c**,**f**) VNA titer at 28 d.p.i. was determined by the rapid fluorescence focus inhibition test, as described in Materials and Methods. Data are expressed as mean ± SEM of international unit (IU). Statistically significant differences between groups are denoted as follows: * *p* ≤ 0.01; ** *p* ≤ 0.005; *** *p* ≤ 0.001.

Current human anti-rabies vaccines are inactivated to improve both the safety for the host and the stability of the preparation. However, multiple doses are required for sufficient immunity. While live-attenuated rabies vaccines are capable of inducing a strong immune response with a single dose, the limitations most often cited for their use are safety and stability. Here we show that IMOVAX^®^, a commercial BPL-inactivated vaccine, either freshly reconstituted or stored at 25 °C or 40 °C for three weeks, is fully protective (100% survival) in immunocompetent mice against an i.m. challenge with the lethal DRV4 virus (Figure 1a). The protection against DRV4 challenge was conferred by the induction of a strong rabies-specific humoral response following immunization with the IMOVAX^®^ vaccine, as evidenced by high levels of circulating IgG antibodies (Figure 1b) and VNAs (Figure 1c). As expected, mice immunized with a fresh preparation of the live-attenuated GG virus resulted in 100% survival after DRV4 challenge, while only 20% of the mice survived when the animals were immunized with the vaccine stored at 40 °C (Figure 1d). However, we found that the live-attenuated GG strain stored at room temperature (25 °C) for three weeks retained sufficient activity to provide full protection against the wild-type DRV4 challenge (Figure 1d). Analysis of the peripheral humoral response of mice infected with GG stored at 25 °C shows that rabies-specific IgGs (Figure 1e) and VNAs (Figure 1f) were both produced. While the VNA titer was lower than that elicited by fresh GG vaccine, it is likely responsible for animal survival, as administration of GG virus stored at 40 °C failed to elicit rabies-specific antibody production (Figure 1e,f) or protect against DRV4 challenge (Figure 1d).

### 3.2. IMOVAX^®^Induces a Mixed Th1/Th2 Immune Response

**Figure 2:**
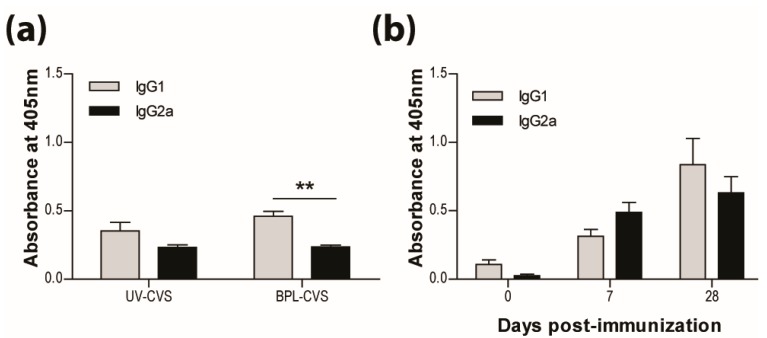
IMOVAX^®^ is protective through the induction of a mixed Th1/Th2 immune response. Virus-specific Ab isotype was determined by ELISA 10 days following immunization with either 1 × 10^5^ f.f.u. of UV- or BPL-inactivated CVS-F3 RABV (**a**) or 0.25 IU of IMOVAX^®^ (**b**). Results are expressed as mean absorbance ±SEM in OD (dilution 1:20). Statistically significant differences between groups (*n* = 5–10 per group) are denoted as follows: ** *p* ≤ 0.005.

In addition to the long-established requirement for VNA, we have previously reported that the bias of the immune response is critical for the clearance of RABV from CNS tissues. We speculate that the bias of the immune response elicited by vaccination is also of importance for long-term protection. As previously reported and seen here, vaccination with RABV inactivated by UV or BPL induces a response producing significantly more IgG1 than IgG2a antibodies (Figure 2a). This is characteristic of a type-2 immune response. We therefore expected the IMOVAX^®^ vaccine, which is BPL-inactivated, to induce the production of IgG1 antibodies. However, both IgG1 and IgG2a RABV-specific antibodies are seen after vaccination with IMOVAX^®^ (Figure 2b), indicating that the response has mixed type-1 and type-2 characteristics.

### 3.3. Addition of Adjuvant to Inactivated Vaccine does not Promote Protection

**Figure 3:**
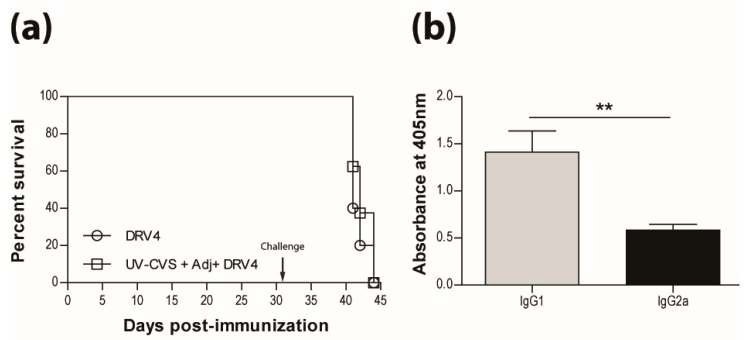
Adding adjuvant to UV-inactivated RABV does not induce protection. (**a**) C57BL/6 mice were immunized with 5 × 10^7^ f.f.u. of UV-CVS-F3 mixed with CFA adjuvant, i.m.g. then challenged 31 days later with 1 × 10^5^ f.f.u. of DRV4 i.n. and monitored for survival. Data are expressed as percent survival. (**b**) Virus-specific Ab isotyping was determined by ELISA 24 days post-immunization. Results are expressed as mean absorbance ±SEM in OD (dilution 1:50). Statistically significant differences between groups *(n* = 10 per group) are denoted as follows: ** *p* ≤ 0.005.

The mixed type-1/-2 response observed following immunization with the IMOVAX^®^ vaccine raises questions about how this inactivated vaccine preparation stimulates Th1 cells. Is there a component with qualities resembling an adjuvant? These immunostimulatory reagents are extensively used for vaccination and have occasionally been reported to promote type-1 immunity. However, we show here that vaccination with UV-inactivated CVS-F3 RABV in Freund’s complete adjuvant (CFA) does not protect mice from subsequent challenge with the lethal DRV4 virus (Figure 3a). The use of CFA in the vaccine evidently did not change the type-2 bias of the response as reflected by the higher levels of RABV-specific IgG1 antibodies produced (Figure 3b).

### 3.4. Vaccination Efficacy is Dictated by the Immunization Regimen and the Challenge Route

**Figure 4:**
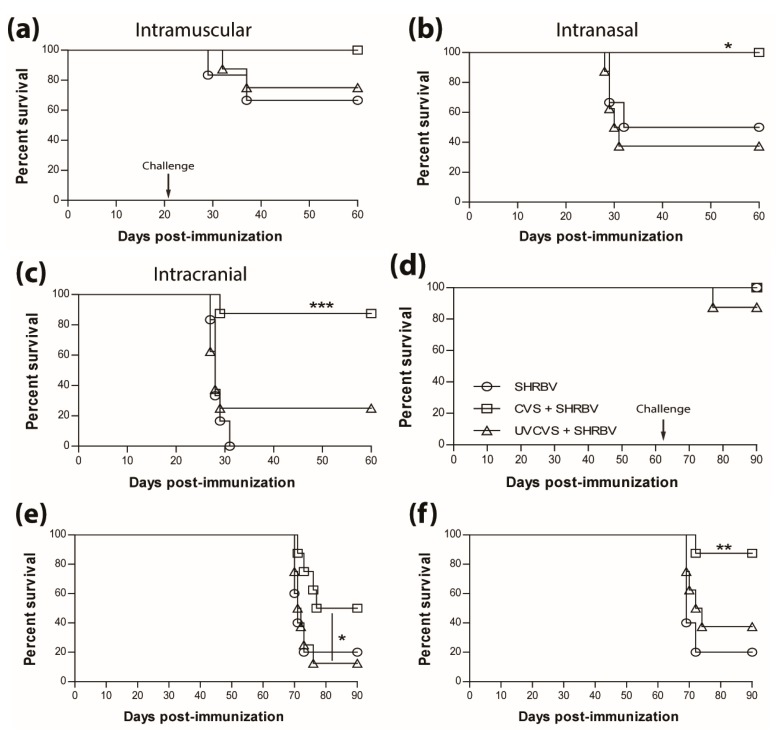
Single immunization with live-attenuated vaccine confer superior long-lasting protection against wild-type virus challenge. Swiss Webster mice were either mock-immunized or immunized with 1 × 10^5^ f.f.u. of live-attenuated CVS-F3 or 1 × 10^8^ f.f.u. of inactivated CVS-F3 (UV-CVS-F3) then challenged 21 (top row) or 63 (bottom row) days later with 1 × 10^4^ f.f.u. of SHBRV-17 virus and monitored for survival. Data are expressed as percent survival (*n* = 10 per group). Animals were immunized either (**a**,**d**) intramuscularly in the gastrocnemius, (**b**,**e**) intranasally or (**c**,**f**) intracranially, then challenged with the corresponding route. Statistically significant differences between groups are denoted as follows: * *p* ≤ 0.01; ** *p* ≤ 0.005; *** *p* ≤ 0.001.

The survival of non-immunized mice infected with the SHBRV-17 RABV differs greatly according to the route of infection, with 33%, 50%, and 100% mortality, for i.m.g (Figure 4a), i.n. (Figure 4b), and i.c. (Figure 4c) routes, respectively. However, regardless of the route of infection, immunization with a single dose of the live CVS-F3 RABV conferred superior protection (90%–100% survival) against SHBRV-17 challenge 21 days later than immunization with the UV-inactivated CVS-F3 (Figure 4, top panels). The difference in protection against i.n. and i.c. immunization/challenge with SHBRV-17 persisted for at least 63 days post-immunization with survival of i.m.g., i.n., and ic. immunized/challenged mice respectively at 100%, 50%, and 87.5% for those vaccinated with live CVS-F3 RABV, versus 87.5%, 12.5%, and 37.5% for animals that had received UV-inactivated CVS-F3 (Figure 4, bottom panel).

### 3.5. Type-1 Immunity is Critical for Protection AgainstWild-Type RABVInfection

**Figure 5:**
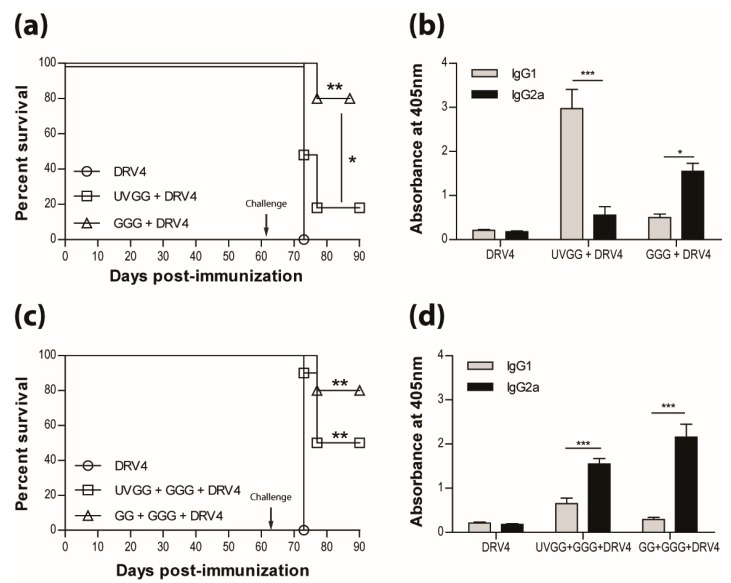
Live-attenuated RABV are protective through the induction of type-1 immunity. C57BL/6 mice were (**a**) immunized with 3 doses of 1 × 10^6^ f.f.u. of UV-GG or 1 × 10^5^ f.f.u. of GGG i.m.g.; (**c**) immunized with 1 × 10^6^ f.f.u. of UV-GG or 1 × 10^7^ f.f.u. of GG followed by a boost of 1 × 10^5^ f.f.u. of GGG 28 days later. All animals were then challenged 63 days later with 1 × 10^5^ f.f.u. of DRV4 i.n. and monitored for survival. Data are expressed as percent survival (*n* = 10 per group). (**b**,**d**) Virus-specific Ab isotyping was determined by ELISA 42 days after immunization. Results are expressed as mean absorbance ±SEM in OD (dilution 1:20). Statistically significant differences between groups (*n* = 10 per group) are denoted as follows: * *p* ≤0.01; ** *p* ≤ 0.005; *** *p* ≤ 0.001.

The results presented above as well as in our previous publications (e.g. reference [15]) indicate that a type-1 immune response is important in dealing with wild-type RABV that reaches the CNS. This is further illustrated in Figure 5a, where immunization with live-attenuated, but not UV-inactivated, RABV efficiently protects against i.n. infection with wildtype DRV4 RABV (80% versus 20% survival). This is despite the production of levels of RABV-specific, type-2-associated IgG1 antibodies in the latter that are higher than the levels of type-1 IgG2a in mice that were vaccinated with the live-attenuated virus (Figure 5b). To determine if the prior induction of a type-2 immune response to RABV interferes with the induction of a more protective type-1 response, mice that received UV-inactivated RABV were boosted with the virus in a live-attenuated format. Survival from an i.n. challenge with DRV4 was improved (from 20% to 50%) but did not reach the level (80%) of mice that were both primed and boosted with live-attenuated RABV (Figure 5c). A change in the bias of RABV-specific serum antibodies from IgG1>IgG2a to IgG2a>IgG1 was seen when animals immunized with UV-inactivated RABV were boosted with the live-attenuated virus (compare Figure 5b,d).

## 4. Discussion

As we previously reported, immunocompetent mice can clear attenuated RABV infection from brain tissues through the local activity of both humoral and cellular type-1 immune mechanisms [14,15,25]. Immune effector delivery across the blood-brain barrier (BBB) proves to be particularly important as this does not happen during wild-type RABV infection [24,26]. IFN-*γ*, a major product of type-1 immunity, is evidently important in RABV clearance from brain tissues through: (i) its induction of type-I interferons that control virus replication [13]; (ii) its contribution to the non-inflammatory changes in BBB function that promote immune cell infiltration [27]; and (iii) its role in driving the local production of VNA [14], which ultimately eliminates the virus from CNS tissues [25]. While the delivery of immune effectors across the BBB is a critical step, the type of immune cells delivered into CNS tissues is also important. CD4^+^ Th2 cells reach CNS tissues during attenuated RABV infection but are evidently non-functional in this environment [15].

The current study reinforces the importance of type-1 immunity in rabies, not merely in the clearance of attenuated RABV but also in the prevention of wild-type RABV infection. Immunization with inactivated RABV induces a type-2 response with high levels of serum RABV-specific antibody but limited protection from an i.n. challenge dose of highly pathogenic DRV4, regardless of whether the inactivated vaccine is administered with CFA. The use of a live vaccine following initial immunization with a killed vaccine induces the production of IgG2a antibodies that are associated with type-1 immunity and improves protection against an i.n. DRV4 challenge, but not to the extent provided by the use a live-attenuated vaccine alone. A comparison of the efficacy of immunization with live-attenuated versus inactivated RABV using the lethal SHBRV provides further insight into the differences in vaccination efficacy. SHBRV administered i.m. in the gastrocnemius is only mildly pathogenic, but lethal for around 80% of mice infected either i.n. or i.c. Infection with the live-attenuated CVS-F3 strain significantly improved survival of mice infected with SHBRV i.n. or i.c., while survival following immunization with inactivated CVS-F3 is only marginally improved in animals challenged i.c. It is also noteworthy that the superior protection against i.n. or i.c. challenge conferred by immunization with live-attenuated virus is consistent, independent of the mouse strain studied (C57BL/6 and Swiss Webster) and the RABV strains used for immunization (GG, GGG, CVS-F3) or challenge (DRV4, SHBRV-17).

Currently, rabies vaccines approved for human use are all produced with inactivated, cell culture-derived RABV. As expected, the commercial IMOVAX^®^ vaccine, consisting of BPL inactivated Pitman-Moore RABV [21], protects 100% of mice against i.m. challenge with DRV4 in the gastrocnemius. While inactivated virus is expected to induce a predominant type-2 response, as shown here for UV-inactivated CVS-F3 and GG as well as beta-propiolactone-inactivated CVS-F3 viruses, IMOVAX^®^ was found to induce the production of RABV-specific antibodies, reflecting a more mixed type-1 plus type-2 response. These data support the predominance of type-1 immunity protection, once triggered, over type-2 immune responses during viral infection. Previously, it has been reported that B cell hybridomas, produced from the peripheral blood mononuclear cells (PBMC) of human donors vaccinated with Rabivac^TM^ (Behringwerke, Marburg, FRG), another vaccine based on inactivated Pitman-Moore RABV, largely elaborate IgG1 and IgG3 rabies VNA [28], reflecting type-1 immunity in humans. This suggests the possibility that either the virus strain or some element of the manufacturing process results in products with some type-1 immunostimulatory properties which are not present in the UV or beta-propiolactone-inactivated viruses studied here. We consider that the capacity to stimulate RABV-specific type-1 immunity is an important basis for an effective rabies vaccine.

Prior work in mice has concluded that adjuvant use with inactivated vaccine enhances immune protection against i.m. challenge with pathogenic RABV [29]. While it is expected that an adjuvant would enhance the immune response, we did not find that the use of CFA with UV-inactivated RABV improved the survival of mice challenged with DRV4 i.n. This suggests that the immune response elicited by inactivated RABV is unable to prevent virus spread in the CNS. It has also been found that rabies immunoglobulin must be administered in conjunction with inactivated vaccine to achieve 100% survival post-exposure [30]. Our finding that live-attenuated RABV vaccines are more effective in the induction of protection against i.n. and i.c. challenge with wild-type RABV provides an explanation as to why GGG [16] and other highly attenuated RABV such as ERAg333 [31] are effective in post-exposure regimens in the absence of added rabies VNA [16]. Infection with GGG would rapidly induce the IFN-*γ*-dependent immune mechanisms capable of preventing wild-type virus replication and trigger the processes that promote immune effector activity in CNS tissues. Thus, treatment with live-attenuated GGG can contain the virus until sufficient antibody production is elicited to mediate virus clearance. As supported by the current data and prior reports of the failure of late stage PEP [9], administration of an inactivated vaccine, and VNA is likely to only be effective if wild-type RABV has not yet reached CNS tissues. Based on the current data, a similar consideration holds for an immunized individual that is exposed to wild-type RABV by a means that introduces the virus in close proximity to the CNS. Pre-existing type-1 rabies immunity would be expected to be protective, but not a type-2 response.

While our data support, in principle, the use of live-attenuated RABV vaccines as alternatives to the inactivated preparations currently used for pre- and post-exposure rabies prophylaxis, there are several considerations for its use. Cost-effectiveness is likely to be excellent as considerably less virus and only a single dose is required to induce strong immunity. However, inactivated RABV are quite stable at different temperatures and live viruses are less so, unless prepared by a vaporization method [32]. Nevertheless, as found in this study, our live-attenuated RABV strain can retain sufficient infectivity to induce fully protective immunity after three weeks at 25 °C without additional processing. In addition to efficacy, safety is the most important prerequisite for the use of a live attenuated virus in vaccination. In this regard, the GGG variant is a good candidate. This vaccine strain was reverse engineered [33] to increase the amount of the immunogenic glycoprotein expressed during infection [34]. GGG has proven nonpathogenic even for developmentally immunocompromised baby mice [16], and does not spread extensively into nervous system tissue [35]. Nevertheless, GGG infection induces immune mechanisms that can clear wildtype RABV from CNS tissues [36] and can be used to establish long-term protection against a wildtype RABV exposure targeting the CNS. Historically, vaccines such as the measles,mumps and rubella (MMR), based on live-attenuated viruses, have proven to be most effective with the benefits far outweighing potential risks. The safety of live-attenuated RABV vaccines in animal models has been well established. For example, GG has been found to be safe and effective in a target species [37]. The transition of live-attenuated RABV vaccines from animals to humans may be driven by the understanding that therapeutic intervention after the virus has entered peripheral nerves requires a type-1 immune response.
